# A Preliminary Exploration Using Imaging Methods to Predict the Possibility of the Recurrence of Serous Ovarian Cancer in Patients Undergoing Total Resection

**DOI:** 10.3389/fonc.2022.754067

**Published:** 2022-04-22

**Authors:** Mengshi Fang, Shan Huang, Jiangning Dong, Hong Yan, Xin Fang, Ping Zhang, Feng Cao, Yulan Chen, Qiujun Zhang

**Affiliations:** ^1^ Department of Radiology, The First Affiliated Hospital of USTC, Division of Life Sciences and Medicine, University of Science and Technology of China, Hefei, China; ^2^ Department of Nuclear Medicine, The Second Affiliated Hospital of Anhui Medical University, Hefei, China; ^3^ Department of Pathology, The First Affiliated Hospital of USTC, Division of Life Sciences and Medicine, University of Science and Technology of China, Hefei, China; ^4^ Department of Medical Oncology, The First Affiliated Hospital of USTC, Division of Life Sciences and Medicine, University of Science and Technology of China, Hefei, China

**Keywords:** ovarian cancer, recurrence, magnetic resonance imaging, diffusion-weighted imaging, apparent diffusion coefficient value, Ki67 proliferation index

## Abstract

**Background:**

The purpose of our research was to explore the value of preoperative CT and MRI examinations and clinical indicators in the prediction of recurrence of ovarian serous carcinoma in patients who underwent satisfactory staging surgery.

**Procedure:**

Detailed inclusion and exclusion criteria were installed to screen all patients collected and the eligible patients were divided into two groups. The CT and MRI features and some clinical characteristics of two groups were analyzed, in addition, the apparent diffusion coefficient (ADC) value in tumor solid region was measured. Univariate analysis was used in this study.

**Results:**

There were 78 patients with histologically proven ovarian serous carcinoma. According to the strict inclusion and exclusion criteria, we retained 29 patients (recurrence group: 11 patients, no recurrence group: 18 patients). For the peritoneal implantation metastasis in CT or MRI images and Ki67 proliferation index (Ki67 PI), the differences between two cohorts were statistically significant (*P* < 0.05). The rate of peritoneal metastasis in the recurrence cohort (10/11, 91%) was higher than that in the no recurrence cohort (7/18, 39%). Patients with high Ki67 PI expression had lower recurrence risk than those with low Ki67 PI expression, HR=0.172 (95%CI: 0.050-0.589, P=0.005), and patients without peritoneal planting had lower recurrence risk than those with it, HR=9.373 (95%CI: 1.194-73.551, P=0.033). For FIGO III patients, ipsilateral fallopian tube involvement was statistically significant between the two groups (*P* < 0.05). The differences in the other preoperative imaging characteristics of ovarian serous cancer, including the volume; capsule of the mass; main components; ADC value; cystic change; bleeding; degree of enhancement of the mainly solid region in 3 periods; and range of tumor involvement in the ovary, uterus, bladder, bowel, and pelvic wall, were not statistically significant. In addition, the differences in the other clinical indicators (i.e., age, FIGO stage) between the two cohorts were not statistically significant.

**Conclusions:**

In CT and MRI examinations before surgery, peritoneal implantation metastasis was suggestive of the possibility of the recurrence of serous ovarian carcinoma in the near future. In addition to that, ipsilateral fallopian tube involvement and Ki67 PI may also indicate the possibility of recurrence (the former was only applicable to FIGO III patients).

## Introduction

Epithelial ovarian carcinoma (EOC) is a malignant tumor with the highest mortality rate among gynecological malignant tumors (1). The clinical symptoms of ovarian cancer are insidious, and the early symptoms are not obvious. Approximately 2/3 of the patients are FIGO III or IV when they visit the doctor for the first time (2). According to the different pathological types of ovarian cancer, the five-year survival rates of these patients with FIGO III and IV were 23.9%~37.0% (2). Serous carcinoma is the most common type of EOC.According to the FIGO Cancer Report 2018, many cases of high-grade serous ovarian carcinoma and peritoneal carcinoma may originate in the tubal umbrella terminus (3). The lethality of EOC is mainly due to the higher risk of recurrence (4, 5). Recurrent ovarian cancers (ROCs) have a very poor prognosis, and most patients with ROC eventually develop resistance to chemotherapy drugs (6). Because of the complexity of the patients’ situation, it is more difficult to choose treatment options for them. The prediction and earlier detection of ROC can help physicians intervene earlier in the treatment process of patients to better achieve individualized and accurate treatments. Imaging examinations can provide important value in the process of follow-up for ovarian carcinoma, but most studies on the risk of recurrence of ovarian carcinoma have mainly focused on clinical and hematological indicators and are relatively lacking in medical imaging data. The purpose of this research was to define the possibility of the use of imaging methods to analyze and predict the recent recurrence of ovarian serous carcinoma, especially for FIGO III patients, with the ultimate hope of helping clinicians to plan more suitable treatments.

## Methods

### Patients and Clinical Information

Upon searching the Hospital Information System (HIS), between August 2015 and September 2019, there were 78 patients who had histologically proven ovarian serous carcinoma in our hospital. We retrospectively searched for these patients in the Picture Archiving and Communication System (PACS), and there were only 46 patients who underwent both pelvic MRI scan and abdominal CT examination before pelvic surgery. The inclusion criteria were as follows: 1) no obvious residual lesions were observed visually after staging operation (the diameter of the residual tumor was ≤1 cm); 2) the time periods between baseline imaging examination and surgery were not more than 7 days; 3) the progression-free survival of the tumor was not less than 6 months; 4) postoperative chemotherapy consisted of 4~6 courses, and the interval of each chemotherapy course was 21 days; 5) patients received first-line chemotherapy approximately 2 weeks after surgery and received first-line chemotherapy for ovarian cancer; 6) the follow-up plan should meet the following requirements: 1 year: once every 3 months, 2-3 years: once every 6 months, >3 years: once a year. Exclusion criteria were as follows: 1) failure to complete adequate and normal chemotherapy or loss to follow-up; 2) severe combined diseases (e.g., heart, lung, liver diseases or renal insufficiency); and 3) ovarian cancer combined with other malignant tumors.

Chemotherapy regimens were mainly included TC (paclitaxel+carboplatin) intravenous chemotherapy, TC+TP (paclitaxel+cisplatin) intraperitoneal intravenous chemotherapy, DC (docetaxel+carboplatin), and PC (cisplatin+cyclophosphamide). Although the longest follow-up time was 40.8 months, in order to avoid the influence of follow-up time on the recurrence of ovarian serous cancer, the follow-up time node was set as 20 months, so that the recurrence of the above patients were divided into two groups, namely the recurrence group and the non-recurrence group. During the follow-up period, patients were required gynecological examinations, ultrasound examinations, and serum tumor marker tests to assess whether the patient had relapsed. When suspicious indications of recurrence were found, patients were advised to have abdominal imaging examinations. Ovarian cancer recurrence was diagnosed when two or more of the following were present: 1) increased levels of tumor markers; 2) a mass found in imaging examination; 3) a mass found on physical examination; 4) hydrothorax and peritoneal fluid; 5) intestinal obstruction of unknown causes.

### CT Examination

CT examinations were carried out on a 64-Row CT scanner (Discovery CT750HD, fifth-generation spectral CT, GE Healthcare), The scanning range was from the upper margin of the diaphragm to the iliac crest. Abdominal plain scan and dynamic enhancement scans of 3 phases (arterial phase, portal phase, delay phase) were adopted. The parameter details were as follows: tube voltage (TV), 120 kV; tube current, 280 mA; frame rotation time, 0.8 s; pitch, 0.984:1; layer thickness, 5.0 mm; and reconstruction interval, 5.0 mm. A double-cylinder power injector (OptiVantage; Mallinckrodt; USA) was utilized to inject the contrast material (4.4 ml/s, 270 mgl/ml, Visipaque, General Electric Healthcare, USA) through peripheral veins.

### MRI Examination

MRI scanning was carried out on a 3.0T system (Signa HDXT, GE Healthcare, USA) equipped with an eight-channel torso array coil. For any larger mass that could not be fully accommodated in an axial plane, a sagittal or coronary scanning sequence was adopted to cover as much of the entire mass as possible. Routine MRI protocols were adopted for the examination of the adnexal lesions, which included axial fast spin echo (FSE) T1-weighted images (T1WI), axial FSE T2-weighted images (T2WI) and fat-suppressed T2WI (FS T2WI). An axial DWI sequence included an echo-planar imaging sequence with an array spatial-sensitivity-encoding technique (ASSET). The specific parameters of T1WI protocol were as follows: repetition time (TR), 500 ms; echo time (TE), 7.8 ms; thickness, 6.0 mm; layer spacing, 2.0 mm; and the number of excitations (NEX), 1. The specific T2WI parameters were TR, 4200 ms; TE, 68 ms; thickness, 6.0 mm; layer spacing, 2.0 mm; and NEX, 2. The parameter details of FS T2WI were similar to those of the T2WI sequence. The parameters of DWI were TR, 4000 ms; TE, 65 ms; thickness, 6.0 mm; layer spacing, 2.0 mm; NEX, 6; and b value = 0 and 1000 s/mm^2^). Liver acquisition with a volume acceleration (LAVA) sequence was adopted for contrast-enhanced pelvic examination, and a power injector (Optistar LE; Mallinckrodt; USA) was performed to inject the contrast material (0.1 mmol/kg, Gadodiamide, General Electric Healthcare, USA). The specific parameters of LAVA MR were TR, 4.5 ms; TE, 1.3 ms; flip angle, 15°; layer spacing, 0 mm; NEX, 1; and band width, 166.67 kHz. The images were obtained from multiple phases after the injection of the contrast agent in the axial and sagittal planes (postcontrast at 20 s, 60 s, 120 s in the axial plane and 150 s in the sagittal plane).

### Radiological Evaluation

MRI and CT images were analyzed carefully on an Advantage Windows workstation 4.5 (AW 4.5 workstation, GE Healthcare, USA) by two radiologists with 31 and 29 years of experience in gynecological imaging. They were blinded to the pathological results, and from each other’s results. The reader variability was evaluated. The average of the measurements for the quantitative data or the outcome of negotiations for the qualitative data acquired by these two radiologists was the final value. For the enrolled patients, the following MRI and CT characteristics were recorded: volume (if there was only one lesion, the maximum transverse diameter plus the maximum vertical diameter plus the maximum anteroposterior diameter/cm; if there were multiple lesions, the maximum lesion size was taken; if multiple lesions showed fusions, the size of all the lesions was taken); whether one or both ovaries was involved (single or bilateral); whether the capsule of mass was intact (yes or no); the presentation of the main components in the mass (type A: mural nodule, type B: solid mass, type C: loose tissue–similar to the spongy appearance); ADC value (Function Tool software was adopted to analyze the ADC value of the tumor, and the ROI was manually placed in the solid area of tumor, which corresponded to high signal intensity; we took 3 measurements at the same level, and the mean value was obtained in mm^2^/s); cystic changes (present or not); the degree of cystic extent (graded as 0-3; grade 0: no sac change; grade 1: the area of cystic changes was less than 1/3; grade 2: 1/3~2/3; grade 3: more than 2/3); bleeding (present or not); the degree of enhancement of the mainly solid region in the arterial period (20 s), venous period (60 s) and the delayed period (120 s~) (mild: less than gluteal muscles; moderate: similar to gluteal muscles; severe: more than gluteal muscles); whether the ipsilateral fallopian tube was involved (yes or no); whether the uterine/bladder/bowel/pelvic wall was involved (yes or no); whether regional lymph node metastasis was found (yes or no, decision criteria: uneven lymph node signal, the disappearance of the lymph node hilum, lymph node short diameter > 1.0 cm); whether there were peritoneal implants; the degree of peritoneal thickening (the thickest point was measured, cm); ascites (present or not); and the deepest diameter of ascites in pelvic MR images (perpendicular to trunk level, cm). The above image indicators were required in the first preoperative examination. During postoperative follow-up, the radiologists judged whether the tumor recurred and the site of recurrence.

### Histopathological Evaluation

All surgical samples were fixed with 4% neutral formaldehyde and embedded with paraffin wax, and 4-µm sections were stained by hematoxylin and eosin (HE) and immunohistochemistry (EliVision). The main antibodies used included Ki67 PI, CAM5.2, EMA, CK7, Ber-EP4, p53, BRCA1, CA-125, WT1, ER, CD99, B72.3, CK20, vimentin, CEA, calretinin, CK5/6, and TTF-1. Antigen negative indicated that all tumor cells were negative, antigen positive indicated that more than 50% of tumor cells were positive, and 0-50% indicated focal positivity or negativity. Histological classification was estimated by two senior pathologists.

### Statistical Analysis

The measurement data with a normal distribution were presented as the “mean ± standard deviation (SD)”, and the independent sample *t* test was used for comparisons between groups. Non-normally distributed measurement data were presented as the median (P25-P75), and we used the rank-sum test (Mann-Whitney U test) for comparisons between the two cohorts. Qualitative materials were statistically described by frequency and percentage (%), and the Fisher exact test was used for comparisons between the two cohorts. Intraclass correlation coefficients (ICCs) were used to evaluate the interobserver agreement in the measurement of volume, ADC values, degree of peritoneal thickening and the deepest diameter of ascites, respectively (ICC > 0.8 was indicative of an almost perfect agreement). Cohen’s Kappa coefficients were used to analyze the consistency of the classification variables, respectively (Cohen’s kappa > 0.8 was indicative of an almost perfect agreement). The test level α was 0.05. A *P* value of < 0.05 was considered statistically significant. SPSS version 20.0 was used to perform all the statistical analyses. The univariate analysis were performed for all parameters between the two groups. Due to the high proportion of FIGO III patients included, the above analysis were also performed for FIGO III patients. In addition, we conducted receiver operating characteristic (ROC) curve analysis on Ki67 PI, and obtained the most optimal cut-off value. Higher than the value was classified as high expression group, lower than the value was classified as low expression group. The survival of the two groups and progression-free survival period (month) were analyzed. Again, all patients were divided into positive and negative groups based on whether peritoneum was implanted and survival curves were analyzed.

## Results

Based on the above inclusion and exclusion criteria, we removed 17 patients. In total, we retained 29 patients for the study (the progression-free survival period ranged from 6 to 40.8 months, with an average of (19.3 ± 9.7) months). All 29 patients underwent laparotomy, and the pathological results of 27 patients were confirmed to be high-grade serous carcinoma, while those of other 2 patients were confirmed to be low-grade serous carcinoma. According to the postoperative follow-up visits, the patients were divided into a recurrence group and a no recurrence group. There were 11 patients in the recurrence group, and 73% (8/11) patients had recurrent lesions in the peritoneum. In the remaining patients, lymph node abnormalities (e.g., inguinal area, adjacent to the iliac vessels, around the rectum, retroperitoneal area, and mesenteric area) were observed. In one patient, both peritoneal and lymph node abnormalities were found. There were 18 patients in the no recurrence group. Statistical analysis results showed that there was a significant difference in Ki67 PI (*P* < 0.05). The result was summarized in **Table 1**.

**Table 1:** The statistical results of univariate analysis of the clinical characteristics of the recurrence and no recurrence groups.

Indicators	Recurrence (n=11)	No recurrence (n=18)	Univariate analysis (*P*)
Age (y)	53.0 ± 7.8	51.6 ± 8.4	0.742
Surgical stage			0.251
FIGO I	1	3	
FIGO II	0	4	
FIGO III	10	11	
Ki67 PI (%)	45.00 ± 24.39	62.78 ± 14.06	**0.045**

Values in bold are statistically significant.

**Figure 1:**
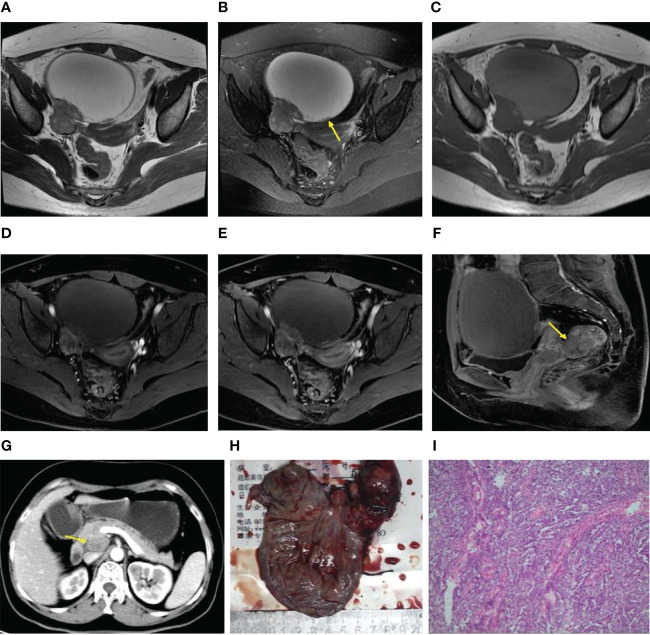
**(A–I)** Axial T2WI and FS T2WI showing a cystic solid mass with a capsule wall nodule [yellow arrow, **(B)**]. T1WI **(C)** displayed high signal in the liquid region of the mass. In the arterial, venous and delayed period, the solid region of the mass revealed severe enhancement, more than the gluteal muscle **(D–F)**. A metastatic nodule was seen in the rectouterine depression [yellow arrow,**(F)**]. Postoperative follow-up revealed a retroperitoneal metastatic lymph node [yellow arrow, **(G)**]. Postoperative gross specimen and HE staining at low magnification showed a high-grade serous carcinoma **(H, I)**.

**Figure 2:**
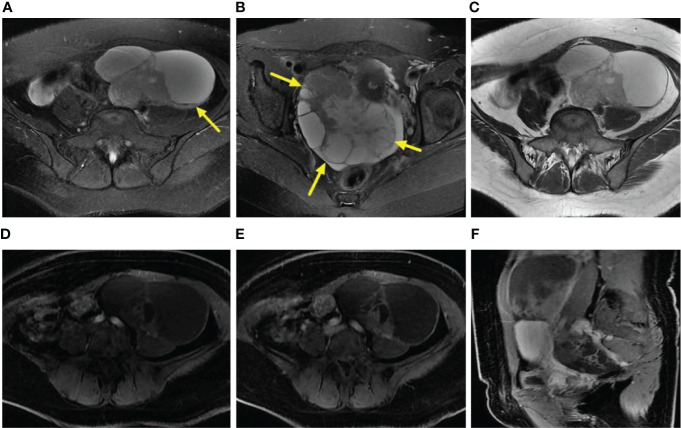
**(A–F)** Axial FS T2WI and T2WI showed multiple cystic solid masses in both ovaries [yellow arrow, **(B)**], and the liquid-liquid plane was visible in part of the capsule [yellow arrow, **(A)**]. In the arterial period, the solid region of the lesion revealed moderate enhancement (i.e., similar to that of the gluteal muscle) **(D)**. In the venous and delayed periods, the solid region of the lesion displayed severe enhancement (i.e., more than that of the gluteal muscle) **(E, F)**.

Regarding the presentation of the main components in the mass, 4 patients were classified as type-A (**Figures 1A, B**), 5 patients as type-B, and 2 patients as type-C. The degrees of cystic extent were as follows: 0 (n=0), 1 (n=4), 2 (n=2), and 3 (n=5) (**Figures 1A–C**). There were 6 patients who displayed high intensity in some cyst regions on T1WI, which may have been bleeding, but this sign did not appear in the remaining 5 patients. In the no recurrence cohort (n=18), there were 8 patients with intact capsules (**Figures 2A–C**). Regarding the presentation of the main components in the mass, 11 patients were classified as type-A (**Figure 2B**), 7 patients as type-B, and 0 patient as type-C. The degrees of cystic extent were 0 (n=1), 1 (n=7), 2 (n=4), and 3 (n=6) (**Figures 2A, B**). There were 7 patients who displayed high intensity in some cyst regions on T1WI that was low intensity on T2WI, indicating a bleeding signal (**Figures 2A, C**), but this feature was not observed in the remaining 11 patients. There were no significant differences in the above indicators (*P*>0.05).

The ADC value of the recurrence group was lower than that of the no recurrence group, but *P*>0.05. On the enhanced images of the arterial period in the recurrence cohort, 3 patients (3/11, 27%) displayed moderate enhancement, and 8 (8/11, 73%) patients displayed severe enhancement (**Figure 1D**), and in the no recurrence group, 5 (5/18, 28%) moderate enhancement (**Figure 2D**), and 12 (12/18, 66%) severe enhancement, *P*>0.05. In the venous period in the recurrence group, 9 (9/11, 82%) severe enhancement (**Figure 1E**), and in the no recurrence group, 16(16/18, 89%) patients severe enhancement (**Figure 2E**), *P*>0.05. In the delayed period, the same manifestation was observed in the recurrence group (**Figures 1F**, **2F**). These findings were shown in **Table 2**.

**Table 2:** The statistical results of the univariate analysis of the ADC values and enhancement degrees of the recurrence and no recurrence groups.

Indicators	Recurrence (n=11)	No recurrence (n=18)	Univariate analysis (*P*)
ADC (×10^-3^mm^2^/s)	0.93 ± 0.27	1.02 ± 0.20	0.353
Arterial period*			1.000
Mild	0	1	
Moderate	3	5	
Severe	8	12	
Venous period*			0.622
Mild	0	0	
Moderate	2	2	
Severe	9	16	
Delayed period*			0.539
Mild	0	0	
Moderate	2	1	
Severe	9	17	

*Enhancement degree.

From the MR imaging findings, in the no recurrence group, there were 8 patients with bilateral ovarian involvement (**Figures 2A, B**). Regarding whether the uterus, bladder, bowel, or pelvic wall was involved, the result was yes in 7 (7/11, 64%) patients in the recurrence group, and the no recurrence group existed 6 (6/18, 33%) cases. However, none of these indicators mentioned above was statistically significant (*P*>0.05).

Lymph nodes were classified as metastatic lymph nodes when there were very typical signs of lymph node metastasis. The results showed that there were 4 (4/11, 36%) patients with lymph node metastasis in the recurrence cohort and 3 (3/18, 17%) patients in the no recurrence cohort (*P*>0.05), but the differences in peritoneal implants were significantly (*P* < 0.05). In the recurrence cohort, 10 (10/11, 91%) patients had peritoneal implants (**Figures 1F–I**), while in the no recurrence cohort, 7 (7/18, 39%) patients had peritoneal implants. With respect to the degree of peritoneal thickening in these two cohorts, the details were summarized in **Table 3** (*P*>0.05).

**Table 3:** The statistical results of univariate analysis of the main CT and MRI features of the recurrence and no recurrence groups.

Indicators	Recurrence (n=11)	No recurrence (n=18)	Univariate analysis (*P*)
Volume (cm^3^)	788.34 ± 646.67	528.32 ± 380.65	0.245
Capsule			0.096
Complete	1	8	
Incomplete	10	10	
Presentation of main components			0.162
A	4	11	
B	5	7	
C	2	0	
Degree of cystic extent			1.000
0	0	1	
1	4	7	
2	2	4	
3	5	6	
Bleeding			0.466
Yes	6	7	
No	5	11	
Ovarian involvement			0.450
Unilateral	4	10	
Bilateral	7	8	
Ipsilateral fallopian tube involvement			0.096
Yes	1	8	
No	10	10	
Uterus/bladder/bowel/pelvic wall involvement			0.143
Yes	7	6	
No	4	12	
The deepest diameter of ascites in pelvic MR images (cm)	4.39 ± 3.27	6.78 ± 4.74	0.121
Lymph node metastasis			0.375
Yes	4	3	
No	7	15	
Peritoneal implants			**0.008**
Yes	10	7	
No	1	11	
Degree of peritoneal thickening (cm)	1.4 (0.5, 2.9)	0 (0, 2)	0.130

Values in bold are statistically significant.

There was a good interobserver agreement between the two radiologists for the above imaging indicators, with ICCs and Cohen’s kappa coefficients ranging within 0.81–0.92 and 0.87–0.99, respectively.

In addition, all indicators were compared between the two groups only for FIGO III patients, and the results were shown in **Table 4**. The results showed that Ki67 PI and ipsilateral fallopian tube involvement were statistically significant between the two groups (*P* < 0.05), while other indicators weren’t statistically significant. These findings were shown in **Table 4**.

**Table 4:** The univariate analysis of the ovarian serous carcinoma on FIGO III between the recurrence and no recurrence groups.

Indicators	Recurrence (n=10)	No recurrence (n=11)	Univariate analysis (*P*)
Age (y)	52.80 ± 8.15	50.73 ± 7.31	0.546
Ki67 PI (%)	44.50 ± 25.65	67.27 ± 11.04	**0.023**
Volume (cm^3^)	824.23 ± 670.64	464.12 ± 336.06	0.150
Capsule			0.586
Complete	1	3	
Incomplete	9	8	
Presentation of main components			0.327
A	4	7	
B	4	4	
C	2	0	
Degree of cystic extent			0.792
0	0	1	
1	3	4	
2	2	3	
3	5	3	
Bleeding			1.000
Yes	5	5	
No	5	6	
Ovarian involvement			0.659
Unilateral	3	5	
Bilateral	7	6	
Ipsilateral fallopian tube involvement			**0.004**
Yes	0	7	
No	10	4	
Uterus/bladder/bowel/pelvic wall involvement			0.659
Yes	7	6	
No	3	5	
The deepest diameter of ascites in pelvic MR images (cm)	4.58 ± 3.38	7.79 ± 5.10	0.104
Lymph node metastasis			0.659
Yes	4	3	
No	6	8	
Peritoneal implants			0.090
Yes	10	7	
No	0	4	
Degree of peritoneal thickening (cm)	1.78 ± 1.20	1.88 ± 1.82	0.883
ADC (×10^-3^mm^2^/s)	0.95 ± 0.27	1.01 ± 0.21	0.604
Arterial period*			1.000
Mild	0	1	
Moderate	3	3	
Severe	7	7	
Venous period*			0.586
Mild	0	0	
Moderate	2	1	
Severe	8	10	
Delayed period*			0.586
Mild	0	0	
Moderate	2	1	
Severe	8	10	

*Enhancement degree. Values in bold are statistically significant.

**Figure 3:**
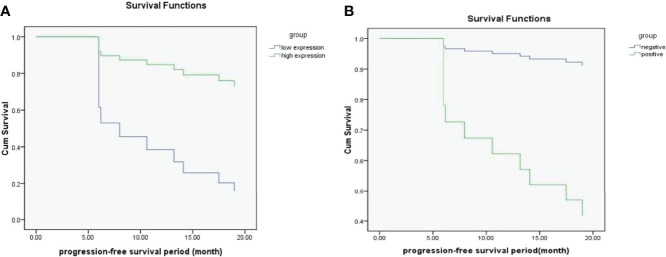
**(A, B)** The survival functions for Ki67 PI low-expression group and high-expression group **(A)**; The survival functions for positive group and negative group (whether peritoneal implantation metastasis was present) **(B)**.

The optimal cut-off value obtained by ROC curve analysis of Ki67 PI was 35% (area under the curve was 0.765, 95% confidence interval(CI) was 0.585-0.945). 35% of Ki67 PI was used as a node to divide all patients into high expression group and low expression group. Survival analysis was conducted on the recurrence status of all patients, and the results were shown in **Figure 3A**. It showed that Ki67 PI was an important factor affecting the recurrence of ovarian serous cancer (P=0.005). Patients with high Ki67 PI expression had lower recurrence risk than those with low Ki67 PI expression, HR=0.172 (95%CI: 0.050-0.589). Similarly, whether peritoneum was implanted that was another important factor affecting the recurrence of ovarian serous cancer (P=0.033), and patients without peritoneal planting had lower recurrence risk than those with it, HR=9.373 (95%CI: 1.194-73.551), and the results were shown in **Figure 3B**.

## Discussion

Ovarian cancer five-year overall survival in early FIGO stages is 90% vs 20–25% in advanced FIGO stages; however, 70% of patients are diagnosed in advanced FIGO stages (7). The high-grade serous subtype accounts for approximately 70% of cases (8). The treatment of ovarian cancer typically includes surgical treatment (staging or debulking) and intraperitoneal, intravenous, or combined chemotherapy (9). There is growing acceptance of the importance of a personalized approach to treatment in patients with ROC and the recognition of the complex interplay between patient- and tumor-related factors that impact the likelihood of patient prognosis (10). If it is possible to predict the likelihood of postoperative recurrence before the first treatment, this may assist in the selection of individualized treatment options. Currently, CA125 and human epididymis protein 4 (HE4) are the only two markers that have been approved by the FDA for monitoring treatment and detecting disease recurrence (7). However, imaging examinations are noninvasive and may be of some benefit to patients if they can help predict the prognosis of ovarian cancer.

Some past studies have suggested that surgical stage, lymph node metastasis, peritoneal implantation, and abdominal water volume played important roles in the prognosis of ovarian carcinoma (11–14). In our study, except for peritoneal implantation, all the other imaging indicators showed no statistical significance. The reasons are analyzed in detail below. First, as for surgical stage, there were more FIGO stage III patients in our study (21/29, 72.4%), while there were fewer FIGO stage I and II patients, which may cause a bias in the results. Second, regarding lymph node metastasis, our study described imaging results to assess lymph node metastasis, and previous studies have focused on the results of operations for lymph node metastasis to predict prognosis. These results may not exactly match, as conventional imaging of lymph node metastasis alone may not be able to assess small lymph node metastasis and distinguish it from lymph node reactive hyperplasia. Third, regarding abdominal water quantity, our study measured the deepest diameter perpendicular to the horizontal axis of the pelvic cavity to represent abdominal water quantity, but the results showed no statistical significance, possibly because the pelvic fluid depth could not completely reflect the total amount of fluid in the entire abdomen or pelvis, and some ascites were scattered in the middle and upper levels of the abdominal cavity. Fourth, regarding peritoneal implantation metastasis, our study suggests that preoperative imaging methods were used to assess the peritoneal metastasis in the recurrence group (10/11, 91%) and the no recurrence group (7/18, 39%), with statistical difference, at the same time, patients with peritoneal planting had higher recurrence risk than those without it. However, peritoneal implants were not statistically significant between the recurrent and non-recurrent FIGO III groups. Because peritoneal implants was histologically confirmed in all FIGO III patients, and most peritoneal implants could be recognized in preoperative imaging evaluation. A few could not be recognized, because the peritoneal implantation foci were very small. So for FIGO III patients, preoperative determination of radiographic implantation metastases in peritoneum should not be used to make inferences about patients’ outcomes. In addition, our study further analyzed the degree of peritoneal thickening, but the results showed no statistical significance. This may imply that no matter how great the degree of peritoneal thickening, the patient’s prognosis was affected as long as there was an implanted metastasis on CT or MRI. An interesting result was that for FIGO III patients, the imaging indicator of ipsilateral fallopian tube involvement was statistically significant between the two groups, and no imaging signs of fallopian tube involvement were observed in the recurrence group. This has not been reported in previous literature, and further study on large sample size or related mechanism is needed. In addition to the above indicators, Ki67 PI was statistically significant whether in large groups or in FIGO III groups, implying that it was not affected by uneven staging. The average Ki67 PI of the recurrence cohort was lower than that of the no recurrence cohort, and there was some overlap between the two groups. Previous studies have yielded mixed results for Ki67 PI for prognostic assessment, so the results require further verification with large sample data in the future.

In our study, the preoperative imaging characteristics of ovarian serous cancer were retrospectively analyzed in detail, including the volume; capsule of the mass; main components; ADC value; cystic change; bleeding; degree of enhancement of the mainly solid region in 3 periods; and range of tumor involvement in the ovary. These imaging characteristics were rarely observed in the past literature. Although the results were not statistically significant, the negative results are of some important clinical significance, namely, that the preoperative imaging characteristics may reflect only the localized characteristics of the tumor, and reliance on preoperative imaging features to estimate the tumor’s recurrence is very difficult. A combination of imaging methods and clinical indicators to evaluate prognosis, is likely to achieve more meaningful results.

Our study had several limitations. First, this study was retrospective, and the number of patients was relatively small. Second, the DWI model adopted in this study was a single index model, which could not eliminate the perfusion related information in ADC values and could not fully reflect the diffusion of pure water molecules inside the tumor. However, intravoxel incoherent motion imaging may provide a more comprehensive analysis of a tissue’s diffusion imaging data. In addition, the follow-up period was short, and the clinical endpoints were not evaluated based on overall survival. These limitations need to be addressed in the future.

In conclusion, in CT and MRI examinations before surgery, peritoneal implantation metastasis was suggestive of the possibility of the recurrence of serous ovarian carcinoma in the near future. In addition to that, ipsilateral fallopian tube involvement and Ki67 PI may also indicate the possibility of recurrence (the former was only applicable to FIGO III patients), but other imaging indexes show no obvious value for indicating the possibility of recurrence.

## Data Availability Statement

The raw data supporting the conclusions of this article will be made available by the authors, without undue reservation.

## Ethics Statement

Written informed consent was obtained from the individual(s) for the publication of any potentially identifiable images or data included in this article.

## Author Contributions

MF: Conceptualization, Methodology, Writing original draft preparation. SH: Software, Data curation, Visualization. J-ND: Supervision, Reviewing. HY: Investigation. XF and FC: Software. PZ and QZ: Validation. YC: Data curation. All authors contributed to the article and approved the submitted version.

## Conflict of Interest

The authors declare that the research was conducted in the absence of any commercial or financial relationships that could be construed as a potential conflict of interest.

## Publisher’s Note

All claims expressed in this article are solely those of the authors and do not necessarily represent those of their affiliated organizations, or those of the publisher, the editors and the reviewers. Any product that may be evaluated in this article, or claim that may be made by its manufacturer, is not guaranteed or endorsed by the publisher.
