# Supplementary figures and images for: A Preliminary Exploration Using Imaging Methods to Predict the Possibility of the Recurrence of Serous Ovarian Cancer in Patients Undergoing Total Resection
Source: Front Oncol. 2022 Apr 22;12:754067. doi: 10.3389/fonc.2022.754067 (PMC9072969; doi:10.3389/fonc.2022.754067)

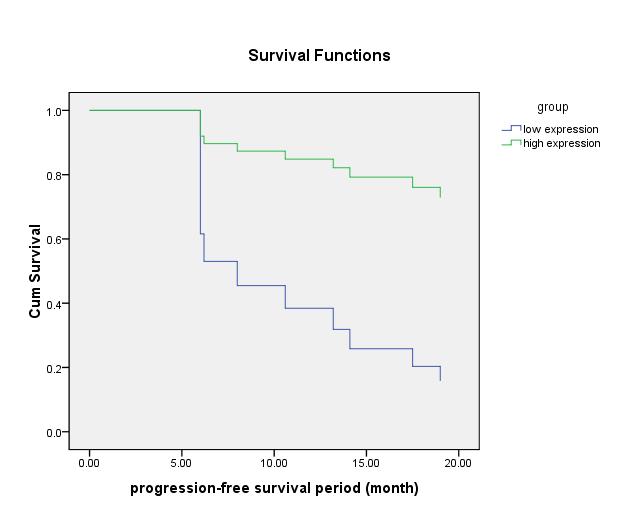

Supplement: Supplementary file 1 [file Image_1.jpeg]

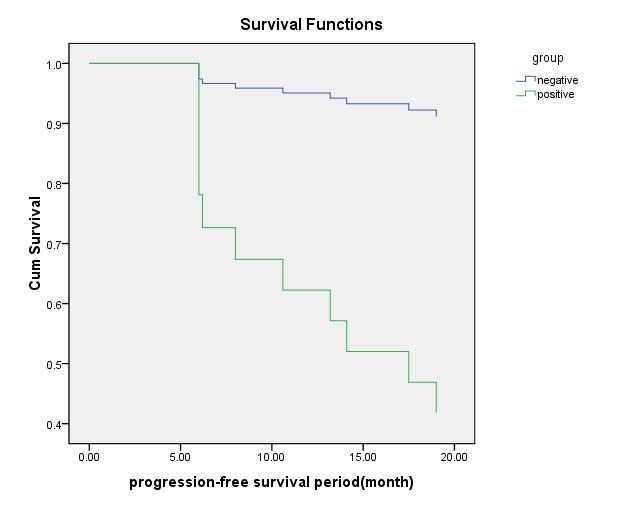

Supplement: Supplementary file 2 [file Image_2.jpeg]
